# Serum MIC-1, LMTK-3 and IGFBP-7 levels in the prognosis of lung cancer treated with ablation combined with chemotherapy

**DOI:** 10.5937/jomb0-62184

**Published:** 2026-04-15

**Authors:** Yilin Fu, Lifeng Shi, Quanxin Lu, Fatao Yu, Hong Xiaofeng, Zeng Guoliang, Meng Li

**Affiliations:** 1 Shandong Provincial Hospital Affiliated to Shandong First Medical University, Department of Thoracic Surgery, Jinan City, China; 2 Shandong First Medical University, Jinan City, China; 3 Anhui Provincial Hospital, Department of Oncology, Hefei City, China

**Keywords:** macrophage inhibitory factor 1, lemur tyrosine kinase 3, insulin-like growth factor, binding protein 7, ablation, advanced lung cancer, makrofag inhibirajući faktor 1, lemur tirozin kinaza 3, protein za vezivanje insulinu sličnog faktora rasta 7, ablacija, uznapredovali karcinom pluća

## Abstract

**Background:**

To explore the value of the serum levels of macrophage inhibitory factor 1 (MIC-1), lemur tyrosine kinase 3 (LMTK-3), and insulin-like growth factor binding protein 7 (IGFBP-7) in the prognosis assessment of patients with advanced lung cancer treated with ablation combined with chemotherapy.

**Methods:**

190 patients with advanced lung cancer who received care at this hospital between January 2022 and June 2024 were chosen to be part of the lung cancer group. The healthy control group consisted of 90 healthy individuals who attended the hospital and underwent physical tests during the same time period. Percutaneous microwave thermal ablation treatment was used to treat every patient with advanced lung cancer, and chemotherapy was administered within one week after treatment. The levels of serum MIC-1, LMTK-3, and IGFBP-7 before and after treatment, and before treatment, were compared between patients with different therapeutic effects in the lung cancer group and the healthy control group. Univariate and multivariate analyses were conducted to examine the factors affecting patient prognosis and the efficacy of serum MIC-1, LMTK-3, and IGFBP-7 levels in predicting patient prognosis in patients with advanced lung cancer.

**Results:**

Serum MIC-1 and LMTK-3 levels in the lung cancer group were substantially higher than those in the healthy control group both before and after therapy (P&lt;0,05). Serum MIC-1 and LMTK-3 levels in the lung cancer group were significantly lower after therapy than before (P&lt;0,05). The serum IGFBP-7 levels in the lung cancer group before and after treatment were significantly lower than those in the healthy control group (P&lt;0,05). After treatment, the serum IGFBP-7 level in the lung cancer group was significantly higher than before treatment (P&lt;0,05). Patients with lung cancer experienced 29 cases of stable response (SD)+ progressive response (PD) and 66 cases of complete response (CR)+ partial response (PR) following treatment. Those with SD + PD advanced lung cancer had significantly higher blood MIC-1 and LMTK-3 levels before treatment than those with CR+PR advanced lung cancer. The serum IGFBP-7 level of advanced lung cancer patients with SD+PD before treatment was significantly lower than that of advanced lung cancer patients with CR+PR. In patients with advanced lung cancer, elevated blood MIC-1 and LMTK-3 levels and lower IGFBP-7 levels before treatment were independent risk factors for death within a year following treatment (P&lt;0,05). The combined detection of serum MIC-1, LMTK-3, and IGFBP-7 levels for predicting death within 1 year after treatment for advanced lung cancer had a sensitivity of 91.3%, a specificity of 95.8%, and an AUC of 0.974. Its AUC was significantly greater than that of MIC-1 (Z=2.378, P=0.017) and LMTK-3 (Z=2.897). The AUC was separately assessed for IGFBP-7 (Z=3.213, P=0.001). However, the AUCs for the three indicators did not differ significantly (P&gt;0.05).

**Conclusions:**

Important markers for assessing the effectiveness of chemotherapy and ablation in the treatment of advanced lung cancer are serum MIC-1, LMTK-3, and IGFBP-7. With the help of combination detection, the prognosis of patients with advanced lung cancer after therapy can be predicted.

## Abstract (sr)

**Uvod:**

Cilj je bio da se istraži značaj nivoa makrofag inhibirajućeg faktora 1 (MIC-1), lemur tirozin kinaze 3 (LMTK-3) i proteina za vezivanje insulinu sličnog faktora rasta 7 (IGFBP-7) u proceni prognoze pacijenata sa uznapredovalim karcinomom pluća lečenih ablacijom u kombinaciji sa hemoterapijom.

**Metode:**

U studiju je uključeno 190 pacijenata sa uznapredovalim karcinomom pluća koji su lečeni u ovoj bolnici između januara 2022. i juna 2024. godine, čineći grupu sa karcinomom pluća. Kontrolnu grupu činilo je 90 zdravih osoba koje su u istom periodu dolazile u bolnicu na preventivne preglede. Svakom pacijentu iz grupe sa karcinomom pluća primenjena je perkutana mikrotalasna toplotna ablacija, a hemoterapija je data u roku od jedne nedelje nakon tretmana. Upoređivani su nivoi serumskog MIC-1, LMTK-3 i IGFBP-7 pre i nakon tretmana, kao i pre tretmana kod pacijenata sa različitim terapijskim efektima u grupi sa karcinomom pluća i u kontrolnoj grupi. Sprovedene su jednofaktorska i višefaktorska analiza kako bi se ispitali faktori koji utiču na prognozu pacijenata i efikasnost nivoa MIC-1, LMTK-3 i IGFBP-7 u predviđanju prognoze kod pacijenata sa uznapredovalim karcinomom pluća.

**Rezultati:**

Nivoi serumskog MIC-1 i LMTK-3 u grupi sa karcinomom pluća bili su značajno viši u odnosu na kontrolnu grupu i pre i posle terapije (P&lt;0,05). Nivoi MIC-1 i LMTK-3 u grupi sa karcinomom pluća bili su značajno niži nakon terapije u odnosu na pre terapije (P&lt;0,05). Nivoi serumskog IGFBP-7 u grupi sa karcinomom pluća pre i posle tretmana bili su značajno niži nego u kontrolnoj grupi (P&lt;0,05). Nakon tretmana, nivo IGFBP-7 u grupi sa karcinomom pluća bio je značajno viši nego pre tretmana (P&lt;0,05). Nakon terapije, kod pacijenata sa karcinomom pluća bilo je 29 slučajeva stabilnog odgovora (SD) + progresivnog odgovora (PD) i 66 slučajeva potpunog odgovora (CR)+ delimičnog odgovora (PR). Pacijenti sa SD+PD imali su značajno više vrednosti MIC-1 i LMTK-3 u krvi pre tretmana u poređenju sa pacijentima sa CR+PR. Nivo serumskog IGFBP-7 kod pacijenata sa SD + PD pre tretmana bio je značajno niži nego kod pacijenata sa CR+PR. Povišeni nivoi MIC-1 i LMTK-3 i niži nivoi IGFBP-7 pre tretmana predstavljali su nezavisne faktore smrtnog rizika u roku od godinu dana nakon terapije kod pacijenata sa uznapredovalim karcinomom pluća (P&lt;0,05). Kombinovana detekcija serumskih MIC-1, LMTK-3 i IGFBP-7 za predviđanje smrti u roku od 1 godine nakon tretmana imala je senzitivnost od 91,3%, specifičnost od 95,8% i površinu ispod ROC krive (AUC) od 0,974. AUC je bio značajno veći u poređenju sa MIC-1 (Z=2,378, P=0,017) i LMTK-3 (Z=2,897). AUC je procenjen posebno za IGFBP-7 (Z=3,213, P=0,001). Ipak, AUC tri indikatora se nije značajno razlikovao (P&gt;0,05).

**Zaključak:**

Serumski MIC-1, LMTK-3 i IGFBP-7 predstavljaju važne markere za procenu efikasnosti hemoterapije i ablacije u lečenju uznapredovalog karcinoma pluća. Kombinovana detekcija ovih markera omogućava predviđanje prognoze pacijenata nakon terapije.

## Introduction

With high incidence and fatality rates, lung cancer is the most prevalent malignant tumour in clinical practice [1]. The number of deaths from lung cancer accounts for the most significant proportion of all malignant tumour deaths, seriously threatening the lives and safety of patients. The vast majority of lung cancers are non-small cell lung cancers [2]. Owing to the lack of typical early symptoms and sensitive diagnostic indicators, these patients are often diagnosed at the middle or advanced stage, which is highly unfavourable to prognosis [3]. Chemotherapy and radiotherapy are important means of treating advanced lung cancer and are economical and practical, but their therapeutic effects are not ideal. Percutaneous microwave thermal ablation therapy is a new treatment method developed over the past few years. It has the advantages of high effectiveness, minimal invasiveness, and few complications, and has been accepted by many patients [4][5][6]. Serological indicators still play irreplaceable roles in the diagnosis, treatment and prognostic assessment of lung cancer. Macrophage inhibitory factor 1 (MIC-1) is a specific marker of lung cancer and a prognostic indicator [7]. One subtype of tyrosine kinase contributes to the transmission of cellular signalling pathways. A cytokine called insulin-like growth factor-binding protein 7 (IGFBP-7) plays a crucial inhibitory role in carcinogenesis and tumour development by preventing the formation of tumour cells [8][9][10].

The optimisation of treatment strategies and improvements in prognosis assessment systems for lung cancer remain key clinical challenges [11][12][13]. In recent years, local ablation techniques combined with systemic chemotherapy have demonstrated potential for synergistic improvement in the comprehensive treatment of advanced lung cancer, notably enhancing local tumour control and extending patient survival [14]. However, the predictive accuracy of current imaging methods and traditional tumour markers for response to combination therapy and long-term outlooks is limited, highlighting an urgent need for more sensitive biological prognostic indicators [15]. Due to their advantages in non-invasive, dynamic monitoring, the use of serum molecular markers has become a crucial breakthrough for predicting treatment response. Studies [16][17][18] indicate that macrophage inhibitory factor-1 (MIC-1) plays a role in regulating tumour inflammatory microenvironments, that lemur tyrosine kinase-3 (LMTK3) influences tumour progression by mediating estrogen receptor signalling, and that insulin-like growth factor binding protein-7 (IGFBP-7) is closely associated with cell proliferation and angiogenesis [19]. The biological functions of these markers in the development, metastasis, and treatment resistance of lung cancer have been gradually understood, but their dynamic changes and prognostic correlations during ablation combined with chemotherapy have yet to be systematically clarified [20].

This study aims to construct a multidimensional prognostic evaluation model by detecting the expression levels of MIC-1, LMTK-3 and IGFBP-7 in the serum of patients undergoing combined ablation and chemotherapy, providing a new basis for individualised efficacy prediction and, at the same time, deepening understanding of the biological response to combined therapy.

## Materials and methods

### General information

Between January 2022 and June 2024, 190 patients with advanced lung cancer, 100 men and 90 women, came to our hospital. These patients were chosen as the lung cancer group. The age ranged from 45 to 75 years, with an average of 61.20±8.97 years. Lesion sites: A total of 108 cases were of the peripheral type, and 82 were of the central type. The tumour types included 86 adenocarcinomas and 104 non-adenocarcinomas. The maximum tumour diameter was >4 cm in 96 patients and ≤4 cm in 94 patients.

Inclusion criteria: Patients with an expected survival period exceeding 3 months and a diagnosis of non-small cell lung cancer confirmed by biopsy under bronchoscopy. Patients must have a Karnofsky functional status score above 60 points.

Exclusion criteria: Patients with concurrent malignant tumours in other parts of the body, incomplete clinical data, coagulation disorders, a history of radiotherapy or chemotherapy, or dysfunction of major organs such as the heart, liver, or kidneys. Also excluded are patients with severe underlying diseases accompanied by intellectual disability or mental illness.

A total of 90 healthy individuals, including 46 males and 44 females, who attended our hospital and underwent physical tests during that time, were selected as the healthy control group. The age ranged from 47 to 77 years, with an average of 60.89±7.11 years. The two groups' baseline data, including age and sex, did not differ significantly (P>0.05) and were comparable. All patients signed the informed consent form, and our hospital's Medical Ethics Committee approved this study (HKYS-2025-A0223).

### Treatment methods

Percutaneous microwave thermal ablation (MWA) was used to treat all patients with advanced lung cancer, and chemotherapy was administered within one week after the procedure. A preoperative routine examination was conducted to determine the puncture site, puncture angle, and needle insertion depth. After satisfactory local anaesthesia, a microwave puncture needle was inserted, and a CT scan was performed to confirm the accurate position of the needle tip. The puncture needle was then secured at the skin entry site, and the microwave tube, along with the normal saline inlet and outlet tubes, was connected for ablation. The procedure generally lasted 12-20 minutes, with a power setting of 30-45 watts. After treatment, a CT reexamination was performed to assess the lesion's residual condition.

### Chemotherapy regimens

Different regimens are used depending on the type of tumour. For patients with adenocarcinoma, a combination of pemetrexed and cisplatin is used for treatment. Cisplatin is intravenously infused at a dose of 75 mg/m^2^ from day 1 to day 3, and pemetrexed at a dose of 500 mg/m^2^ is intravenously infused on day 1. The gemcitabine-cisplatin (GP) regimen was adopted for treatment. Cisplatin was intravenously infused at a dose of 75 mg/m^2^ on days 1 to 3. On the 1st and 8th days, gemcitabine was intravenously infused at a dose of 1,000 mg/m^2^. One treatment cycle lasts for 21 days, and four consecutive cycles constitute one course of treatment.

### Therapeutic effect evaluation

After the patient's treatment course was completed, the therapeutic effect was evaluated. If all visible tumours disappeared and clinical symptoms disappeared for at least 4 weeks, complete remission (CR) was achieved. A partial response (PR) was defined as a reduction of more than 50% in the tumour lesion volume, disappearance of clinical symptoms for more than 4 weeks, and no new lesions. The tumour lesion shrinks by 25%-50%, and no new lesions appear, indicating stable disease (SD). A reduction of less than 25% in tumour volume, or an increase in tumour size, is considered progressive disease (PD).

### Follow-up investigation

After treatment, the patients were followed up through outpatient reexaminations, WeChat, QQ, and phone calls. The follow-up period ended on July 30, 2022. All patients were followed for more than 1 year, and death was the endpoint. In the first year following therapy, the patient was checked every 3 months, and in the second year, every 6 months.

### Blood specimens

After patient admission and completion of one course of chemotherapy, approximately 5 mL of venous blood was collected from the elbow. The blood was allowed to stand at room temperature for about 20 minutes, then centrifuged at 3,000 rpm for 10 minutes with a 15 cm radius rotor. Approximately 3 mL of the supernatant was collected and stored at -80°C for subsequent testing. Serum levels of MIC-1, LMTK-3, and IGFBP-7 were measured using enzyme-linked immunosorbent assay (ELISA), following the manufacturer's instructions (Mike Bio Co., Ltd).

### Observation indicators

Changes in the levels of serum MIC-1, LMTK-3, and IGFBP-7 were observed in the lung cancer and healthy control groups. Changes in serum MIC-1, LMTK-3, and IGFBP-7 levels were compared in patients with advanced lung cancer before and after treatment, and in patients with different therapeutic outcomes. Univariate and multivariate analyses of factors affecting prognosis were conducted. To analyse the efficacy of serum MIC-1, LMTK-3 and IGFBP-7 levels before treatment in predicting mortality after treatment for advanced lung cancer.

### Statistical processing methods

Data processing and statistical analysis were conducted using SPSS 22.0. x̄±s is used to represent data with uniform variance and a normal distribution. Analysis of variance was used to compare groups; the SNK-q test was used to compare groups pairwise; and the independent-samples t-test was used to compare two independent samples. The 2 test was used to compare groups, and count data are presented as percentages or counts. The variables affecting the prognosis of patients with advanced lung cancer were examined using multivariate logistic regression. The effectiveness of each index in predicting individuals with advanced lung cancer within a year was investigated using a receiver operating characteristic (ROC) curve. P values less than 0.05 were regarded as statistically significant.

## Results

### Changes in the levels of serum MIC-1, LMTK-3 and IGFBP-7 in the two groups

The lung cancer group's blood MIC-1 and LMTK-3 levels were statistically significantly (P<0.05) higher than those of the healthy control group both before and after treatment. Serum MIC-1 and LMTK-3 levels were significantly lower in the lung cancer group than before treatment (P<0.05). The serum IGFBP-7 levels in the lung cancer group before and after treatment were significantly lower than those in the healthy control group (P<0.05). After treatment, the serum IGFBP-7 level in the lung cancer group was significantly higher than before treatment (P<0.05; see Table 1).

**Table 1:** Changes in serum levels of MIC-1, LMTK-3, and IGFBP-7 in two groups (x̄ ± s).

Group	n	MIC-1 (pg/mL)	LMTK-3 (ng/mL)	IGFBP-7 (ng/L)
Healthy control group	90	378.42±98.51	5.42±1.25	57.22±7.13
Lung cancer group	190			
Before treatment		1909.59±589.68	16.41±4.79	24.54±5.15
After treatment		1242.32±234.21	8.82±2.17	39.52±6.28

### Comparison of serum MIC-1, LMTK-3 and IGFBP-7 levels before treatment in patients with advanced lung cancer with different therapeutic effects

After the course of treatment, 66 cases of CR+PR and 29 instances of SD+PD occurred in patients with advanced lung cancer. Patients with SD+PD had considerably higher serum MIC-1 and LMTK-3 levels than those with CR+PR before therapy, and their serum IGFBP-7 levels were significantly lower than those of CR+PR patients (P<0.05), see Table 2.

**Table 2:** Serum MIC-1, LMTK-3, and IGFBP-7 levels in patients with advanced lung cancer with varying therapeutic outcomes before treatment.

Efficacy	n	MIC-1 (pg/mL)	LMTK-3 (ng/mL)	IGFBP-7 (ng/L)
CR+PR	132	1643.22±412.87	14.39±3.41	26.82±3.77
SD+PD	58	2515.80±473.06	21.02±4.31	19.37±4.00
t		9.069	8.041	8.714
P		<0.001	<0.001	<0.001

### Univariate analysis of prognosis in patients with advanced lung cancer

All patients were successfully followed up for more than one year. Among them, 23 died, and 72 survived. Among the deceased patients, the percentages of patients with adenocarcinoma and those with a maximum tumour diameter larger than 4 cm. Additionally, the pretreatment blood MIC-1 and LMTK-3 levels were much greater than those of the survivors. The difference was statistically significant (P<0.05). Compared with the surviving patients, the deceased patients' pretreatment serum IGFBP-7 level was much lower. The difference was statistically significant (P<0.05), see Table 3.

**Table 3:** Univariate analysis of prognosis in advanced lung cancer patients (n).

Indicators	Death (n=46)	Survival (n=144)	x^2^/t	P
Age (years)			0.275	0.600
>60	22	82		
≤60	24	62		
Gender			0.593	0.441
Male	20	80		
Female	26	64		
Location of lesion			0.077	0.781
Surrounding type	24	84		
Central type	22	60		
Family history of cancer			0.182	0.669
Yes	10	42		
No	36	102		
Tumour type			8.586	0.003
Adenocarcinoma	34	52		
Non-adenocarcinoma	12	92		
Maximum diameter of tumour (cm)			10.859	0.001
>4	38	58		
≤4	8	86		
MIC-1 (pg/mL)	2530.77±511.48	1711.16±463.01	7.205	<0.001
LMTK-3 (ng/mL)	20.36±4.41	15.15±4.21	5.113	<0.001
IGFBP-7 (ng/L)	19.44±4.48	26.17±4.21	6.577	<0.001

### Prognostic multivariate analysis in individuals with advanced lung cancer

As independent variables, the indicators that showed statistically significant differences in the single-cause analysis were given values: tumour type (=1 adenocarcinoma, adenocarcinoma =0), tumour diameter (>4 cm =1, 4 cm =0 or less), serum MIC-1 (>1, 909.59 pg/mL=1, 1 or less). Multivariate logistic regression analysis was performed on 909.59 pg/mL=0), LMTK-3 (>16.41 ng/mL=1, ≤16.41 ng/mL=0), and IGFBP-7 (>24.54 ng/L=1, ≤24.54 ng/L=0). Increased levels of serum MIC-1 and LMTK-3 and decreased levels of IGFBP-7 before treatment were found to be independent risk factors for death within one year after treatment in patients with advanced lung cancer (P<0.05), see Table 4.

**Table 4:** Multivariate prognostic analysis for individuals with advanced lung cancer.

Indicators	β	SE	Wald χ^2^	P	OR	OR95%CI
Tumour type	1.940	1.877	1.069	0.301	6.961	0.176~275.502
Maximum tumour<br>diameter	-2.362	1.917	1.519	0.218	0.094	0.002 4.033
MIC-1	0.004	0.001	9.436	0.002	1.004	1.002~.007
LMTK-3	0.289	0.116	6.177	0.013	1.335	1.063~1.676
IGFBP-7	-0.329	0.123	7.190	0.007	0.719	0.565~0.915

### Efficacy of serum MIC-1, LMTK-3 and IGFBP-7 levels before treatment in predicting death after treatment in patients with advanced lung cancer

Binary logistic regression analysis was conducted on the indicators based on whether death occurred, and the equation

Y=0.004xXMIC-1+0.29xXLMTK-3-0.35XXIGFBP-7-7.77

was obtained and used as the combined detection index. The combined detection method's sensitivity for predicting post-treatment death in individuals with advanced lung cancer was 91.3%, the specificity was 95.8%, and the AUC was 0.974. The AUC was significantly greater than that of MIC-1 (Z=2.378, P=0.017), LMTK-3 (Z = 2.897, P=0.004), and IGFBP-7 (Z = 3.213). When the three indicators were examined independently, there was no statistically significant difference in AUC (P>0.05; see Table 5).

**Table 5:** Efficacy of serum MIC-1, LMTK-3, and IGFBP-7 levels in predicting mortality after treatment of advanced lung cancer.

Indicator	Truncation value	Sensitivity (%)	Specificity (%)	AUC	95%CI
MIC-1	2096.75 pg/mL	82.6	80.6	0.883	0.801~0.940
LMTK-3	19.79 ng/mL	65.2	91.7	0.801	0.707~0.876
IGFBP-7	23.60 ng/L	95.7	73.6	0.875	0.791~0.934
MIC-1+MTK-3 + IGFBP-7	-	91.3	95.8	0.974	0.919~0.996

## Discussion

In clinical practice, the primary treatments for patients with advanced lung cancer include chemotherapy, radiation, and targeted therapy [21][22]. However, long-term use can lead to drug resistance and complications in patients, affecting their prognosis. In recent years, microwave thermal ablation therapy has been used to treat various tumours, achieving good results. It causes minor trauma, can relieve patients' symptoms, prolong their survival period, and thereby improve their prognosis [23][24][25]. Clinically, the prognostic assessment of patients with advanced lung cancer relies mainly on imaging examinations. However, owing to their high cost and limitations, such as radiation exposure, short-term repeated examinations are not feasible. Serological indicators are highly valuable for evaluating the therapeutic effect in malignant tumours, and they offer advantages such as high repeatability, rapid detection speed, and convenient sampling. They have gradually been applied to the clinical assessment of malignant tumour prognosis and have become a hot topic in the evaluation of malignant tumours [26].

The lung cancer group's serum MIC-1 level was much higher before treatment than it was in the healthy control group, and it was significantly lower following chemotherapy and ablation, indicating that the source of MIC-1 may be closely related to lung cancer [27]. In the early stage of cancer, it mainly induces tumour apoptosis and inhibits tumour proliferation, whereas in the late stage, it promotes tumour cell metastasis and contributes to tumour progression [28]. The results of this study revealed that the serum MIC-1 level in SD+PD patients with advanced lung cancer was significantly greater than that in CR+PR patients with advanced lung cancer. Moreover, the proportion of patients with a maximum tumour diameter >4 cm and the MIC-1 level of deceased patients with advanced lung cancer were significantly greater than those of surviving patients (P<0.05). Serum MIC-1 levels might be an essential indicator of the prognosis of patients with advanced lung cancer [29][30][31]. Serum MIC-1 is expressed at low levels in healthy individuals, whereas its serum level is transiently elevated in patients with acute inflammation. In patients with mid-stage cancer, the level is continuously elevated. Research [32] has shown that serum MIC-1 levels are a valuable biomarker for predicting the prognosis of patients with malignant tumours. When the serum MIC-1 level before treatment was 2,096.75 pg/mL. In patients with advanced lung cancer, the sensitivity was 82.6%, the specificity was 80.6%, and the AUC was 0.883 for predicting death within a year following treatment [33].

After treatment, the serum LMTK-3 level was significantly lower than before, indicating a specific association between LMTK-3 and lung cancer. LMTK-3 is a subtype of tyrosine kinase. The transduction of signalling pathways affects the biological characteristics of cells. The serum LMTK-3 level before treatment in patients with advanced lung cancer treated with SD+PD was significantly greater than that in patients with advanced lung cancer treated with CR+PR. Moreover, the serum LMTK-3 level before treatment was significantly higher in patients who died within one year after treatment than in surviving patients. Estrogen generally increases gene transcription via the estrogen receptor (ER) pathway, promoting cell proliferation and apoptosis. LMTK-3 regulates the ER pathway by promoting ER gene activation. Research has shown that the ER pathway is widely present in target organs such as the lungs and stomach. In particular, the biological characteristics of lung cancer patients are closely associated with ER status. Therefore, the activation of the ER pathway is one of the reasons for the high expression of LMTK-3 in the cancer tissues and serum of patients with lung cancer. When the serum LMTK-3 concentration was 19.79 ng/mL before treatment, the sensitivity for predicting death within one year after treatment in patients with advanced lung cancer was 65.2%, the specificity was 91.7%, and the AUC was 0.801, indicating that the serum LMTK-3 concentration before treatment may be an indicator for predicting death within one year in patients with advanced lung cancer [34].

After treatment, the serum IGFBP-7 level in lung cancer patients increased significantly. It mainly inhibits tumour growth by regulating cell proliferation, invasion and angiogenesis. In malignant tumours, IGFBP-7 gene expression is inhibited substantially, thereby promoting tumour angiogenesis. IGFBP-7 mainly prevents the degradation of IGF-1 by related hydrolases by competitively inhibiting the binding of IGF-1 to its receptor [35]. The serum IGFBP-7 level of advanced lung cancer patients receiving a therapeutic effect of SD+PD was significantly lower than that of advanced lung cancer patients receiving a therapeutic effect of CR+PR. Moreover, the serum IGFBP-7 levels of deceased patients were substantially lower than those of surviving patients. Reduced blood IGFBP-7 levels were found to be an independent risk factor for death within a year following treatment for advanced lung cancer, according to multivariate analysis. Patients with advanced lung cancer who received a therapeutic effect of SD+PD had a considerably lower serum IGFBP-7 level than those who received a therapeutic benefit of CR+PR. Moreover, the serum IGFBP-7 levels of deceased patients are significantly lower than those of surviving patients [36]. According to multivariate analysis, a reduction in serum IGFBP-7 levels was a risk factor for death within a year following advanced lung cancer treatment. When the blood IGFBP-7 level was 23.6 ng/L before treatment, the study's findings showed that the sensitivity, specificity, and AUC for predicting death within a year following treatment in patients with advanced lung cancer were 95.7%, 73.6%, and 0.875, respectively.

Moreover, in this study, logistic regression was conducted on MIC-1, LMTK-3, and IGFBP-7, and the resulting equations were used as the joint detection model. The sensitivity of combined detection in predicting death within one year after treatment in patients with advanced lung cancer was 91.3%, the specificity was 95.8%, and the AUC was 0.974. Its AUC was significantly greater than those of MIC-1, LMTK-3 and IGFBP-7, which were detected separately. This indicates a certain complementarity among the three indicators. The specific internal connection remains unclear. The ER and IGF-1 signalling pathways mutually interfere with and synergistically promote lung cancer development. Therefore, the particular mechanism still needs further study.

## Conclusion

Serum levels of MIC-1, LMTK-3, and IGFBP-7 are essential indicators of treatment efficacy after ablation combined with chemotherapy for advanced lung cancer. Combined detection helps forecast the prognosis of patients with advanced lung cancer after treatment.

## Dodatak

### Conflict of interest statement

All the authors declare that they have no conflict of interest in this work.

## References

[1] Meyer M, Fitzgerald B G, Paz-Ares L, Cappuzzo F, Jänne P A, Peters S, Hirsch F R (2024;Aug 24). New promises and challenges in the treatment of advanced non-small-cell lung cancer. Lancet.

[2] Greer J A, Post K E, Chabria R, Aribindi S, Brennan N, Eche-Ugwu I J, Halpenny B, Fox E, Lo S, Waldman L P, Pintro K, Rabideau D J, Pirl W F, Cooley M E, Temel J S (2024;Oct 20). Randomized Controlled Trial of a Nurse-Led Brief Behavioral Intervention for Dyspnea in Patients With Advanced Lung Cancer. J Clin Oncol.

[3] Sen T, Takahashi N, Chakraborty S, Takebe N, Nassar A H, Karim N A, Puri S, Naqash A R (2024;Aug). Emerging advances in defining the molecular and therapeutic landscape of small-cell lung cancer. Nat Rev Clin Oncol.

[4] Reuss J E, Rosner S, Levy B P (2024;Jun). Antibody drug conjugates in advanced lung cancer: Is this a new frontier?. Clin Adv Hematol Oncol.

[5] Wu L, Li X, Yan J (2024;Jul). Commentary: Machine learning developed an intratumor heterogeneity signature for predicting prognosis and immunotherapy benefits in cholangiocarcinoma. Transl Oncol.

[6] Xue H, Chen Y, Zhou Y (2024;Dec 6). Radioimmunotherapy: A game-changer for advanced non-small cell lung cancer. Front Immunol.

[7] Loh J, Low J L, Sachdeva M, Low P Q J, Wong R S J, Huang Y, Chia P L, Soo R A (2023;Jul-Dec). Management of Oncogene Driven Locally Advanced Unresectable Non-small Cell Lung Cancer. Expert Rev Anticancer Ther.

[8] Solta A, Ernhofer B, Boettiger K, Megyesfalvi Z, Heeke S, Hoda M A, Lang C, Aigner C, Hirsch F R, Schelch K, Döme B (2024;Feb 24). Small cells - big issues: Biological implications and preclinical advancements in small cell lung cancer. Mol Cancer.

[9] Tsukada A, Morita C, Shimizu Y, Uemura Y, Naka G, Takasaki J, Nokihara H, Izumi S, Hojo M (2024;Sep). Efficacy of first-line immune checkpoint inhibitor and anti-angiogenic agent combination therapy for Kirsten rat sarcoma viral antigen-mutant advanced non-small-cell lung cancer: A systematic review and network meta-analysis. Thorac Cancer.

[10] Wu L, Yang L, Qian X, Hu W, Wang S, Yan J (2024;Aug 16). Mannan-Decorated Lipid Calcium Phosphate Nanoparticle Vaccine Increased the Antitumor Immune Response by Modulating the Tumor Microenvironment. J Funct Biomater.

[11] Yu L, Wang R, Zhao Y, Wu Y, Wang L, Chen H, He Z, Wang Q, Wu Y (2023;Jan-Dec). Advances in the Treatment of Rare Epidermal Growth Factor Receptor Mutations in Advanced Nonsmall-Cell Lung Cancer. Technol Cancer Res Treat.

[12] Wu L, Zheng Y, Liu J, Luo R, Wu D, Xu P, Wu D, Li X (2021;Apr 20). Comprehensive evaluation of the efficacy and safety of LPV/r drugs in the treatment of SARS and MERS to provide potential treatment options for COVID-19. Aging (Albany NY).

[13] Kundu M, Dey A, Dasgupta S (2025;Jun). Replication stress response and radioresistance in lung cancer: Mechanistic insights and advanced therapeutic approaches. Curr Probl Cancer.

[14] Melosky B, Vincent M D, McGuire A L, Brade A M, Chu Q, Cheema P, Martins I, Spicer J D, Snow S, Juergens R A (2024;Sep 15). Modern era systemic therapies: Expanding concepts of cure in early and locally advanced non-small cell lung cancer. Int J Cancer.

[15] Lee S, Park S, Lee H Y, Jeon H, Lee S, Choi S, Eo W (2023;May-Jun). A potential treatment option for advanced non-small cell lung cancer: Three cases. Explore (NY).

[16] Wu L, Zhong Y, Wu D, Xu P, Ruan X, Yan J, Liu J, Li X (2022;Sep 10). Immunomodulatory Factor TIM3 of Cytolytic Active Genes Affected the Survival and Prognosis of Lung Adenocarcinoma Patients by Multi-Omics Analysis. Biomedicines.

[17] Gridelli C, Attili I, Bennati C, Bironzo P, Bria E, Cortinovis D L, Soto P H, de Marinis F (2025;Apr). Immunotherapy in advanced non-small cell lung cancer: What to do for the 'Invisible' patients after IPSOS trial results?. Lung Cancer.

[18] Simone C B 2nd (2024;Nov 1). Enhancing Outcomes in Locally Advanced Non-Small Cell Lung Cancer Through Stereotactic Dose Escalation. Int J Radiat Oncol Biol Phys.

[19] Buma A I G, Schuurbiers M M F, van Rossum H H, van den Heuvel M M (2024). Clinical perspectives on serum tumor marker use in predicting prognosis and treatment response in advanced non-small cell lung cancer. Tumour Biol.

[20] Wu L, Liu Q, Ruan X, Luan X, Zhong Y, Liu J, Yan J, Li X (2023;Jun 29). Multiple Omics Analysis of the Role of RBM10 Gene Instability in Immune Regulation and Drug Sensitivity in Patients with Lung Adenocarcinoma (LUAD). Biomedicines.

[21] Opalikhin A, Friedland S, Madariaga M L, Owen D H, Besse B (2025;Jun). Surgical and Perioperative Advances for Patients With Locally Advanced Non-Small Cell Lung Cancer. Am Soc Clin Oncol Educ Book.

[22] Lobinger D, Hiebinger A, Eicher F, Groß G, Shalabi I, Reiche A, Bodner J (2023;Dec). Rescue surgery in palliative indication as last therapeutic option for complicated advanced stage lung cancer. Eur J Surg Oncol.

[23] Wu L, Zheng Y, Ruan X, Wu D, Xu P, Liu J, Wu D, Li X (2022;Jan 1). Long-chain noncoding ribonucleic acids affect the survival and prognosis of patients with esophageal adenocarcinoma through the autophagy pathway: Construction of a prognostic model. Anticancer Drugs.

[24] Khan S R, Breadner D (2025;Mar 31). Unveiling the Synergistic Potential: Bispecific Antibodies in Conjunction with Chemotherapy for Advanced Non-Small-Cell Lung Cancer Treatment. Curr Oncol.

[25] Kearns E, Chu A K, Nissim R, Wheatley P P, Aubry T, Lebel S (2024;Nov). The Supportive Care Needs of Individuals Living With Advanced or Metastatic Lung Cancer Receiving Targeted or Immunotherapies. Psychooncology.

[26] Wu L, Zhong Y, Yu X, Wu D, Xu P, Lv L, Ruan X, Liu Q, Feng Y, Liu J, Li X (2022;Oct 1). Selective poly adenylation predicts the efficacy of immunotherapy in patients with lung adenocarcinoma by multiple omics research. Anticancer Drugs.

[27] Durbin L, Murali B, Li S, Hawthorne S, Clark O (2024). Treatment patterns in advanced/metastatic non-small cell lung cancer in China: results from the Cancer-MPact® survey 2021. Future Oncol.

[28] Gautam R P, Reingold D, Pathak N, Verma S, Gupta A, Meti N, Molto C, Malik P S, Linford G, Mittal A (2025;Aug 9). Recent Advances in the Management of EGFR-Mutated Advanced Non-Small Cell Lung Cancer: A Narrative Review. Curr Oncol.

[29] Wu L, Li H, Liu Y, Fan Z, Xu J, Li N, Qian X, Lin Z, Li X, Yan J (2024). Research progress of 3D-bioprinted functional pancreas and in vitro tumor models. International Journal of Bioprinting.

[30] Zhang Y R, Lu Y H, Lin C M, Ku J W (2025;Aug). Pretreatment CT Texture Analysis for Predicting Survival Outcomes in Advanced Nonsmall Cell Lung Cancer Patients Receiving Immunotherapy: A Systematic Review and Meta-Analysis. Thorac Cancer.

[31] 31. *** (2024;Oct). Deaths from advanced lung cancer have dropped significantly since immunotherapy became standard-of-care. Saudi Med J.

[32] Wu L, Li X, Qian X, Wang S, Liu J, Yan J (2024;Feb 12). Lipid Nanoparticle (LNP) Delivery Carrier-Assisted Targeted Controlled Release mRNA Vaccines in Tumor Immunity. Vaccines (Basel).

[33] Roberts H N, Solomon B, Harden S, Lingaratnam S, Alexander M (2024;Jul). Utility of 30-Day Mortality Following Systemic Anti-Cancer Treatment as a Quality Indicator in Advanced Lung Cancer. Clin Lung Cancer.

[34] Moretti M, Wellekens S, Dirkx S, Vekens K, van Laethem J, Ilsen B, Vanderhelst E (2023;Feb). Features of post-obstructive pneumonia in advanced lung cancer patients, a large retrospective cohort. Infect Dis (Lond).

[35] Chen S, Wang C, Huang T, Tsai J, Wang H, Yen Y, Tseng Y, Wu C, Chang J, Shiau A (2024;Sep 23). Advancing Lung Cancer Treatment with Combined c-Met Promoter-Driven Oncolytic Adenovirus and Rapamycin. Cells.

[36] Sato S, Sezaki R, Shinohara H (2024;Aug). Significance of preoperative evaluation of modified advanced lung cancer inflammation index for patients with resectable non-small cell lung cancer. Gen Thorac Cardiovasc Surg.

